# Label-Free Imaging and Biochemical Characterization of Bovine Sperm Cells

**DOI:** 10.3390/bios5020141

**Published:** 2015-04-01

**Authors:** Maria Antonietta Ferrara, Giuseppe Di Caprio, Stefano Managò, Annalisa De Angelis, Luigi Sirleto, Giuseppe Coppola, Anna Chiara De Luca

**Affiliations:** 1 Institute for Microelectronics and Microsystems, National Research Council, Via P. Castellino, 111, 80131 Naples, Italy; E-Mails: dicaprio@rowland.harvard.edu (G.D.C.); luigi.sirleto@cnr.it (L.S.); giuseppe.coppola@cnr.it (G.C.); 2 Rowland Institute, Harvard University, Cambridge, MA 02142, USA; 3 Institute of Protein Biochemistry, National Research Council, Via P. Castellino, 111, 80131 Naples, Italy; E-Mails: s.manago@ibp.cnr.it (S.M.); a.deangelis@ibp.cnr.it (A.D.A.)

**Keywords:** spermatozoa, biophotonics, sex-identification, digital holography microscopy, Raman spectroscopy, Raman imaging

## Abstract

A full label-free morphological and biochemical characterization is desirable to select spermatozoa during preparation for artificial insemination. In order to study these fundamental parameters, we take advantage of two attractive techniques: digital holography (DH) and Raman spectroscopy (RS). DH presents new opportunities for studying morphological aspect of cells and tissues non-invasively, quantitatively and without the need for staining or tagging, while RS is a very specific technique allowing the biochemical analysis of cellular components with a spatial resolution in the sub-micrometer range. In this paper, morphological and biochemical bovine sperm cell alterations were studied using these techniques. In addition, a complementary DH and RS study was performed to identify X- and Y-chromosome-bearing sperm cells. We demonstrate that the two techniques together are a powerful and highly efficient tool elucidating some important criterions for sperm morphological selection and sex-identification, overcoming many of the limitations associated with existing protocols.

## 1. Introduction

In order to upgrade animal production management systems as well as genetic improvement programs, artificial insemination (AI) is widely used. Indeed, AI allows controlling the genetic quality of breeding herds and preselecting the sex of offspring. Sperm morphology assessment is one of the most important criteria for determining the quality of the semen sample: semen with high levels of morphological sperm abnormalities could reflect genital dysfunction, and it is one of the most common factors of male infertility; therefore, it is not recommended for AI [1,2]. Besides, application of AI using semen with preselected sex could have several potential benefits as: higher production levels with reduced costs, improvement in animal health and welfare, reduction of environmental impact due to the elimination of the unwanted sex before they grow to adulthood, and faster genetic progress [3].

Among farm animals, in this work the attention was focused on bull. Considering that cattle producers are interested in identifying the most healthy and fertile bulls, semen quality is examined with several methods [4], with particular care taken for sperm with intact acrosomes, sperm with normal morphology [5,6,7,8] and sex sorted [3].

For this reason, there is a growing interest to study the spermatozoa morphological alterations and, control and preselect the sex of progeny by identifying X- and Y-bearing sperm cells. To obtain such information, the used techniques have to be: (i) label-free, to reduce costs and exclude all unwanted effects that may be introduced by tags; (ii) non-destructive, to avoid any vitality alteration of the analyzed sperm; (iii) independent on the experience of the technician and environmental conditions (such as, temperature, pH level, and duration). With this aim, the optical approaches are thoroughly investigated [9].

A quantitative morphological characterization of bull sperm cells can be performed by optical microscopy, but it could be difficult and time-consuming. Thus, a fine z-scanning of the biological sample is required in order to acquire and collect different focal planes, and then the acquired images are used in post-elaboration to produce a 3D image of the object under investigation. Detailed morphological information can be achieved by using an atomic force microscopy (AFM) [10,11]. However, the required sample preparation and the effective cost of AFM instruments makes its use in animal production industry not promising.

Holography presents new opportunities for studying cells and tissues non-invasively, quantitatively and without the need for staining or tagging. Digital holography (DH) has been successfully employed to perform morphological analysis on bovine sperm cells [12,13,14]. It is important pointing out that in DH 3D image is obtained from the reconstruction of a single acquired hologram, without any mechanical scanning. This technique has the great benefit to manage quantitative information and allows carrying out different numerical analysis (such as, estimation area, profiles along particular directions, and selection of different zones) that can help understanding the link between the abnormal morphology and the male infertility [9,12].

Another important parameter to consider in sperm selection is the sex-sorting. Several approaches have been proposed in literature to separate X-and Y-bearing sperm cells: fluorescence-activated flow cytometer and cell sorting (FACS) [15,16,17], immunological [18], dimensional assessment [19,20], and electrophoretic separation [21]. However, some of these techniques require external labeling that can kill/damage cells, or reduce significantly sperm motility, while others need a fixed sample, hampering the study of native cells.

The possibility to overcome these limitations is a focus of relatively recent research and great efforts are devoted to the study of a new sorting system that allows an efficient non-invasive characterization and keeps cells alive after sorting. Raman Spectroscopy (RS) could be the perfect candidate as it is a non-invasive and very specific technique providing the biochemical analysis of cellular components with a spatial resolution in the sub-micrometer range [22,23,24]. This technique offers a huge potential for solving biomedical problems, such as single-cell characterization [25], biomarkers detection [26] and sex-identification for instance [3], as demonstrated by the recent increase in this field publications [25,26,27,28]. Moreover, when RS is combined with a microscope, it allows the reconstruction of high contrast bio-chemical maps of the investigated sample [29,30,31,32].

In this paper, morphological and biochemical investigation of single bovine sperm cells is performed by DH and RS. Concerning morphological aspects, comparing the results of these two techniques an intriguing correlation between the amount of protein and the presence of a “protuberance” is pointed out. Concerning sex-sorting aspects, we demonstrate that a purely morphological sorting based on volume determination of X- and Y-bearing sperm cells is not enough, while higher accuracy is provided by biochemical assessment.

## 2. Theoretical Background

### 2.1. Digital Holography

DH is a powerful technique based on interference between two laser beams commonly named reference beam and object beam (that illuminates the object under test). The incoming light is scattered by the biological object forming the complex object wavefield:

(1) $$O\left(x,y\right)=\left|O(x,y)\right|e^{i\varphi (x,y)}$$

where |O| is the amplitude and φ is the phase, and x and y denote the Cartesian coordinates in the recording plane (hologram plane). The phase φ(x, y) is related to the optical path difference (OPD) that depends on the refractive index and thickness both of the biological sample and the material containing the object itself [33]:

(2) $$∆\varphi \left(x,y\right)=\frac{2\text{π}}{\lambda }OPD$$

where a transmission configuration has been considered. Due to their different optical path, both waves interfere at the surface of the recording device, and the hologram is proportional to the intensity of this interference pattern. The phase of the object wavefield is encoded in the intensity fringe pattern adding the reference beam R(x,y) = |R(x,y)| e^iφ^_R_^(x,y)^.

In DH, an image sensor (CCD or CMOS) acquires the hologram; *i.e.*, a 2D rectangular raster of M × N pixels, with pixel pitches ∆x and ∆y in the two directions [34,35]. Then, by a mathematical analysis, is possible to obtain the complex field of the object beam [36]. In particular, the hologram is 2D Fourier transformed. In order to spatially separate the first-order diffraction from the whole spatial frequency spectrum, a small angle has been introduced between the two interfering beams in our setup (off-axis configuration). Thus, in order to retrieve the full-field distribution of the object beam, the first-order diffraction is bandwidth filtered and shifted to the origin of the k-space [37]. Since the whole field is known, it is possible to reconstruct the optical wavefront at different distances from the plane of acquisition applying the Fourier formulation of the Fresnel-Kirchhoff diffraction formula [38].

Each value of the matrix forming the DH microscopy phase map represents the phase delay of the light passing through the sample, which is connected with the thickness of the cell. This kind of information is purely quantitative and can be simply used for the morphological analysis of the cells and for the selection of the anomalies.

### 2.2. Raman Spectroscopy

RS is a light scattering technique. When monochromatic radiation interacts with the sample, most photons are elastically scattered (Reyleigh scattering); therefore, they have the same energy as the incident photons. However, a small fraction (approximately one in one million) is scattered at optical frequency different from the frequency of the incident photons. The process leading to this inelastic scattering is known as Raman effect. The difference in energy between the incident and the scattered photon, or Raman shift, corresponds to the energy of a vibration of the scattering molecule:

(3) $$\bar{\nu }=\frac{1}{{\text{λ}}_{incident}}-\frac{1}{{\text{λ}}_{scattered}}$$

The origin of the modified frequencies found in Raman scattering is explained in terms of energy transfer between the scattering system and the incident radiation. Therefore, a Raman band is to be characterized not by its absolute wavenumber but by the magnitude of its wavenumber shift $\bar{\text{ν}}$. A plot of intensity of scattered light *versus* energy difference is a Raman spectrum. Raman scattering can occur with a change in vibrational and rotational energy of the molecule. Because the energy levels are unique for every molecule, a Raman spectrum provides a “fingerprint” of the sample, allowing identification of unknown sample components to a degree that is unmatched by other techniques [39,40,41,42,43].

Additionally, by combining RS with a microscope it is possible to reconstruct the distribution of assigned chemicals within a cell. This kind of application is usually referred as Raman imaging or mapping [29,30,31,32]. Raman image can be generated by raster scanning the sample through the laser probe and collecting a spectrum at each position. Finally, the collected data can be processed to generate false color images based on the sample biochemical composition [42,43].

## 3. Experimental Set Up

**Figure 1:**
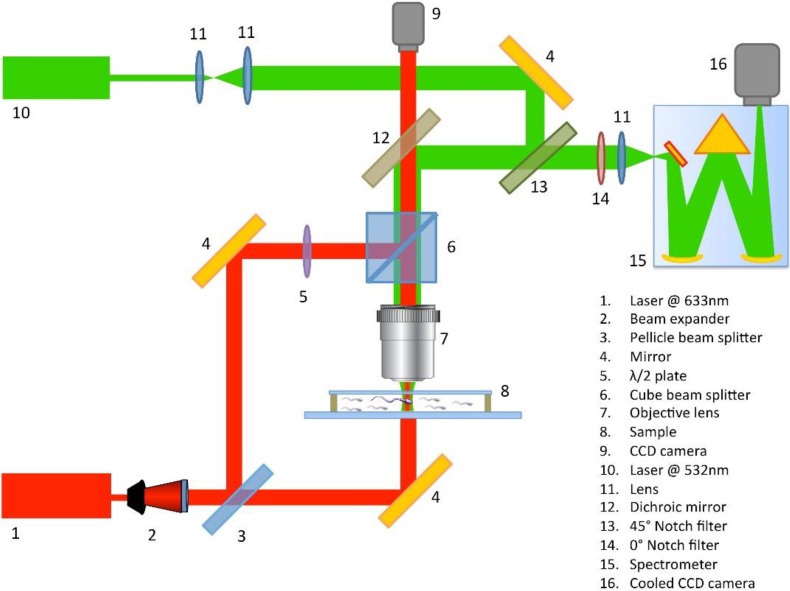
Innovative experimental set up that brings together digital holography and Raman spectroscopy for full label-free characterization of biological samples.

Figure 1 shows the main components of our experimental set up which essentially consists of a DH system combined with a Raman spectrometer.

The laser used to record the holograms was a He-Ne emitting at λ = 633 nm. It was filtered and expanded, and then split into reference and object beam by a pellicle beam splitter (optic component n.3 in Figure 1). The object beam, after the sample illumination, was collected through a microscope (Objective lens 40×, 0.65 N.A.) and recombined to the reference beam by a second beam splitter (optic component n.6 in Figure 1). Finally, the generated holographic pattern was projected onto a CCD camera (1392 × 1040 pixels array; each pixel had dimension Δx = Δy = 4.7 mm).

In our experiments, a double exposure technique is used. The first exposure is made on the sample under investigation, while the second one is made on a reference surface in proximity of the object. Information about all the aberrations introduced by the optical components, including the defocusing due to the microscope objective, is incorporated into the second acquired hologram. In such a way, it is possible to compensate these aberrations by numerically manipulating the two holograms.

Raman spectra were excited using a second laser emitting at λ = 532 nm. It was initially expanded and mixed to the holographic beams though a dichroic mirror (optic component n.12 in Figure 1), reflecting the radiation at 532 nm and being transparent in the spectral range around 633 nm. The microscope used for the DH was used to focus the Raman light on the sample (objective lens 100×, oil immersion, 1.30 N. A.), probing a sample volume about 0.3 µm^2^ × 0.8 µm. The laser power on the sample was set to 5 mW to avoid any possible photodamage. Back scattered light from the sample, was collected and collimated by the same objective lens following back the same path as the holographic beams, was reflected by the dichroic mirror, finally reaching a notch filter (optic component n.13 in Figure 1). This last reflected the Rayleigh scattered light while provided a good transmission of the inelastically scattered Raman light. The Raman light is filtered using a laser-blocking filter to eliminate the residual Rayleigh scattering and then focused onto the entrance slit of the monochromator, set at 100 μm. The monochromator is equipped with an 1800 lines mm^−1^ holographic grating providing an estimated spectral resolution of approximately 2 cm^−1^. The Raman scattered light is finally detected by using a back-illuminated CCD, thermoelectrically cooled at −70 °C.

### Sample Preparation

The bovine sperm cells to be analyzed were prepared by the Institute “Lazzaro Spallanzani” after fixation in suspension of the seminal material with 0.2% glutaraldehyde solution in phosphate buffered saline (PBS) without calcium and magnesium (1:3 v/v). A drop with volume 4 μL has been deposed in the sample chamber. The sample chamber used in our experiments was made by using an 80 μm deep vinyl spacer between a quartz slide (1 mm in thickness) and a quartz coverslip (150 μm in thickness) (UQG). The coverslip has been linked to the slide by means of a strip of varnish. The cells were allowed to sediment (for 30 min) on the quartz coverslip before starting the experiments.

## 4. Results and Discussion

**Figure 2:**
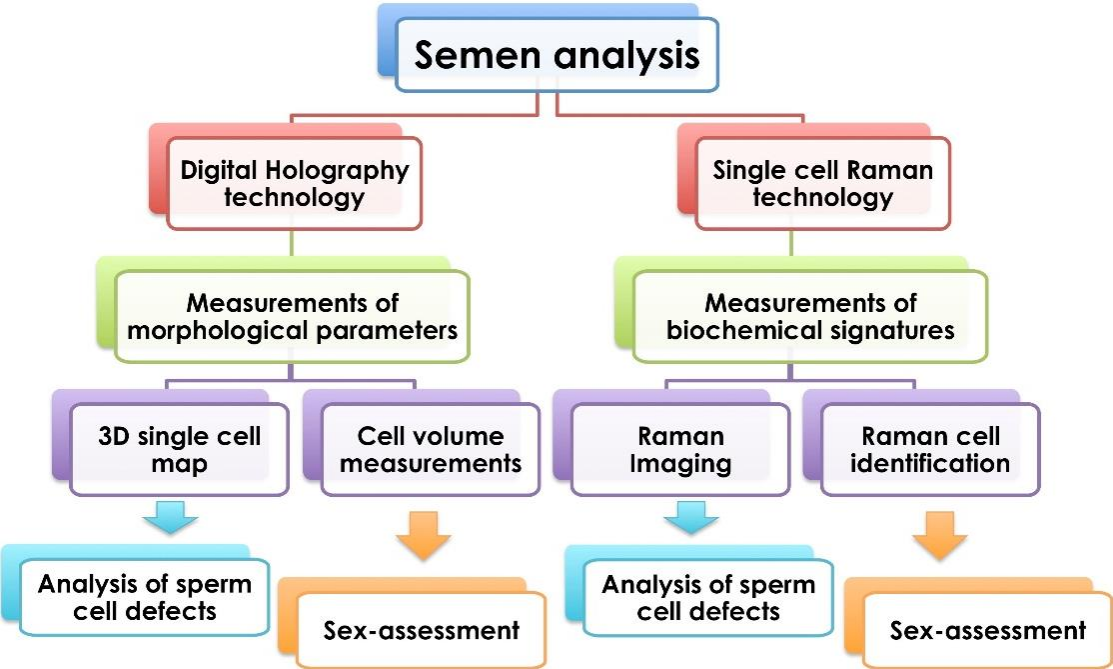
Overview of the single sperm cell analysis technology. Single cells can be analyzed by Digital Holography (DH) or Raman spectroscopy (RS). DH microscopy based on morphological parameters measurement is a fast and label-free technology allowing the reconstruction of 3D maps of single selected cells and measurements of cell volumes/thickness. RS detects biomolecule vibrations from a single cell, which serves as a cellular intrinsic “fingerprint”. It is a sensitive and label-free technology allowing the production of pseudo-color images according to the Raman spectral band intensities and the identification of cell phenotype and physiological state. Both technologies can be applied to analyze sperm cell defects or characterize X- and Y-bearing sperm cells.

In order to focus on the most important parameters measured in a semen analysis, such as the morphology, biochemical alterations and sex-assessment of the sperm, we performed the comparative analysis summarized in Figure 2.

### 4.1. Morphological Characterization of Bull Sperm Cells

**Figure 3:**
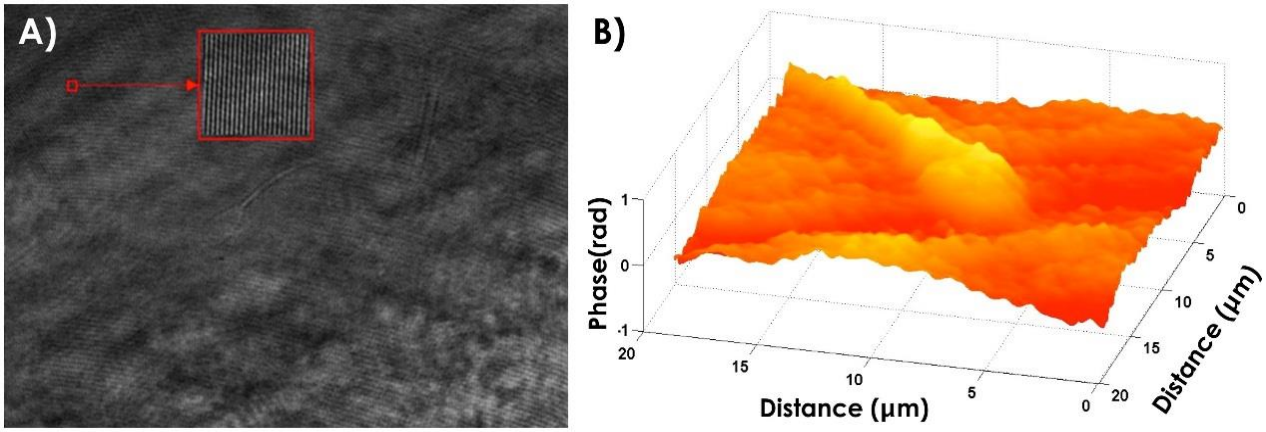
(**A**) Acquired hologram, a region is enhanced in order to show the interference pattern (inset); (**B**) Pseudo 3D representation of the phase map of a bovine spermatozoon obtained by digital holography microscopy.

The morphology of bovine sperm cells and the integrity of their structures were investigated by DH microscopy. An example of an acquired hologram is reported in Figure 3A, where the inset shows the intensity of the fringe pattern due to the superimposition of the object and reference beams. The reconstructed three-dimensional shape of a bovine spermatozoon is reported as a pseudocolor plot in Figure 3B. Quantitative information about the thickness of the sample under test can be obtained by Equation (2), by the phase-contrast map of the sample and considering a uniform refractive index of about 1.35, as reported in literature [33].

The DH microscopy approach provides a 3D reconstruction of the sample from a single acquired image (*i.e.*, the hologram), without any mechanical scanning.

High-resolution images of spermatozoa morphological alteration have been obtained employing this method, and some common defects were characterized in our previous papers. For example, in Refs. [9,12], the reconstructed images of a spermatozoon with a cytoplasmatic droplet along the tail, a bent tail sperm cell and a sperm with broken acrosome are reported.

**Figure 4:**
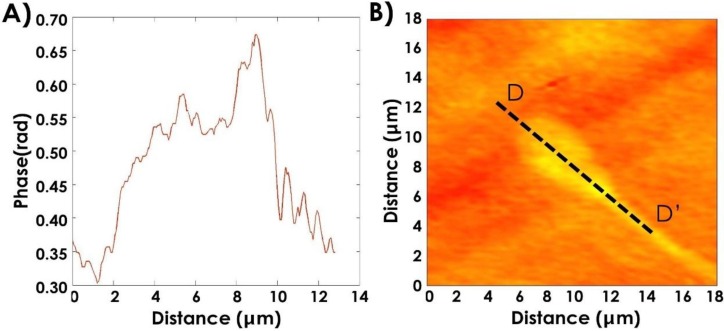
(**A**) Profile of the head along the line DD' illustrated in (**B**).

Figure 3B shows an interesting feature, common to many analyzed spermatozoa: a “protuberance” on the post-acrosomal region of the head. This alteration is well visible in the profile reported in Figure 4A relative to the line DD' shown in Figure 4B and it has been highlighted for the first time in our previous paper [12]. Among possible explanations of the “protuberance” nature, an artifact due to a possible different refractive index between the post-acrosomal region, (containing almost exclusively highly compact chromatin) and the acrosome should be excluded. Thus, the same characteristic has been observed in images acquired by confocal dual core confocal microscope (DCM) 3-D Leica microscope and reported in our previous paper [12]. However, both holographic and confocal images only reveal the presence of morphological defect, while they are not able to identify its biochemical constitution. A first explanation for the estimated area and volume variation is that the “protuberance” highlighted by DH microscopy could be due to the presence of the centrioles into the structures connecting the tail to the head.

**Figure 5:**
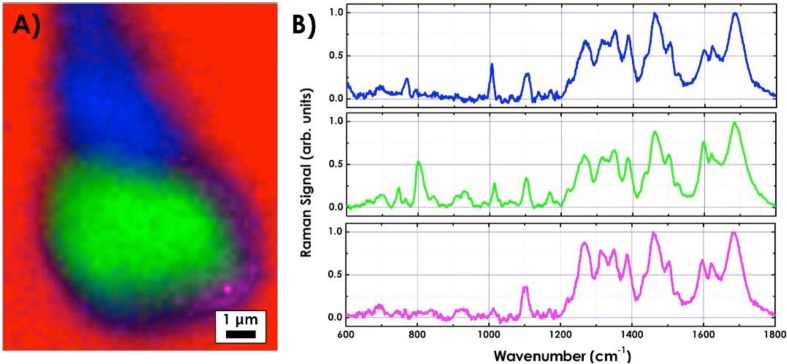
(**A**) Raman image (9 × 12 µm^2^) of a spermatozoon acquired on Xplora inverted Raman microscope of HORIBA Jobin Yvon; (**B**) Raman spectra (integration time: 10 s) acquired from tail (blue line), nucleus (green line) and acrosomal vesicle (magenta line). The colors of the spectra correspond to the colors in the image.

To obtain complementary information on the biochemical structure of spermatozoa, Raman imaging technology is used. Figure 5A shows a 9 × 12 μm^2^ Raman image of a spermatozoon acquired by raster scanning the sample under the microscope (mapping step 0.2 μm). Spectra were recorded at each image pixel with an integration time of 10 s at a laser power of 15 mW measured at the microscope objective. Figure 5B shows the Raman spectra acquired from distinct region of the spermatozoon. The nucleus Raman spectrum (green line) is characterized by strong Raman bands assigned to the nucleic acids and DNA (726, 785, 1095 and 1581 cm^−1^). Pronounced bands corresponding to proteins and lipids (1200–1300, 1480 and 1600–1680 cm^−1^) identify the acrosomal vesicle spectrum (magenta line). Finally, the tail Raman spectrum (blue line) is characterized by a sharp peak around 751 cm^−1^ previously assigned to mitochondria and strong peaks at 1005 and 1450 cm^−1^ consistent with the presence of proteins in this spectral region. The band assignment of the Raman peaks is summarized in Table 1.

**Table 1:** Raman peaks assignment of sperm cell spectra.

Raman peak assignment			
Raman band (cm^−1^)	Nucleic acids	Protein	Lipids
**726**	A		
**751**	U, C		
**785**	T, C, bk		
**831**	OPO	Tyr	
**1005**		Phe	
**1095**	PO_2_^-^		C–C
**1255**		Amide III	=CH
**1290**	A, T	Amide III	=CH
**1320–1380**	A, G	CH def	CH_3_, CH_2_
**1450–1480**	A, G	CH_2_	CH_2_
**1575–1585**	A, G		
**1600–1700**	T	Amide I	C=C

Abbreviations: C, Cytosine; T, Thymine; A, Adenine; G, Guanine; U, Uracil are DNA/RNA bases; Phe, Phenylalanine; Tyr, Tyrosine.

**Figure 6:**
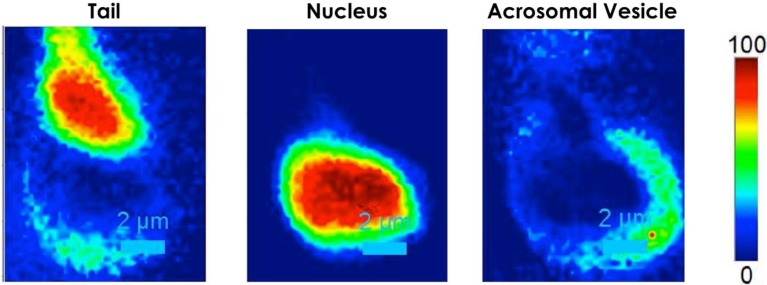
2D intensity map of each Raman spectrum corresponding to different regions of the spermatozoon: tail, nucleus and acrosomal vesicle.

By assigning a specific color to each of the described spectra, the false color image of the spermatozoon can be reconstructed. As for the DH, the Raman image reveals the “protuberance” on the post-acrosomal region of the head. This feature was visible only for a few (around 2%–3%) out of the dozens of analyzed cells. The protuberance can be better identified from the 2D intensity map reported in Figure 6, clearly showing a local concentration of material in the sperm region connecting the tail to the head. A detailed inspection into Figure 5B (blue line) demonstrated that the sharp vibrations originating from the presence of proteins in the spectral region at 1005 and 1450 cm^−1^, clearly less intense in the other spectra, together with the presence of mitochondria, correspond to the most important Raman markers of such “protuberance”. Our results suggest an intriguing correlation between the amount of protein and the presence of the “protuberance” that could be associated with the presence of centrioles in the sperm region connecting the tail to the head. Indeed, the centrioles are cylindrical cell structures composed essentially by tubulin, therefore affecting the local concentration of protein. However, in order to give a correct biological explanation to the origin of this formation, additional analyses and comparisons with traditional fluorescence confocal microscopy results are required.

### 4.2. Characterization of X-and Y-Bearing Sperm Cells

Holographic imaging sex-sorting is entirely based on a possible difference in sperm head size and volume due to the variation in the amount of DNA, as the X-chromosome being larger than the Y-chromosome. As reported in Section 2.1, using DH it is possible to reconstruct the phase map of an object under test and from Equation (2) its thickness. For this reason, this technique can be used to evaluate the sperm head volume in order to achieve sex-sorting.

**Figure 7:**
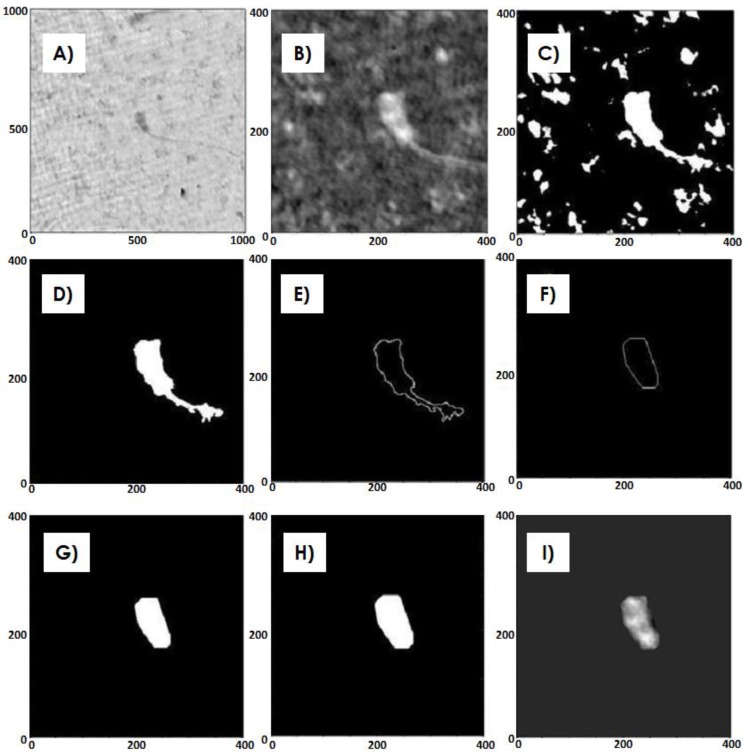
(**A**) Phase map; (**B**) Cut phase map; (**C**) Otsu’s method; (**D**) Regionprops maximum area; (**E**) Boundary; (**F**) Polygonal interpolation; (**G**) Filling; (**H**) Expansion; (**I**) Product phase-mask.

With this aim, a MATLAB code was implemented, and the applied procedure is reported in Figure 7. On the zoomed phase map (Figure 7A,B), retrieved by means of the aforementioned numerical approach, the threshold is computed by applying the Otsu’s method (Figure 7C) [44]. Then, the “regionprops”, a MATLAB function, is applied to measure properties of image regions (Figure 7D), and boundary of the region of interest and its polygonal interpolation are performed (Figure 7E,F). A filling and an expansion of the selected area are done (Figure 7G,H); finally the product between the phase map (Figure 7B) and the obtained mask (Figure 7H) is implemented (Figure 7I).

**Figure 8:**
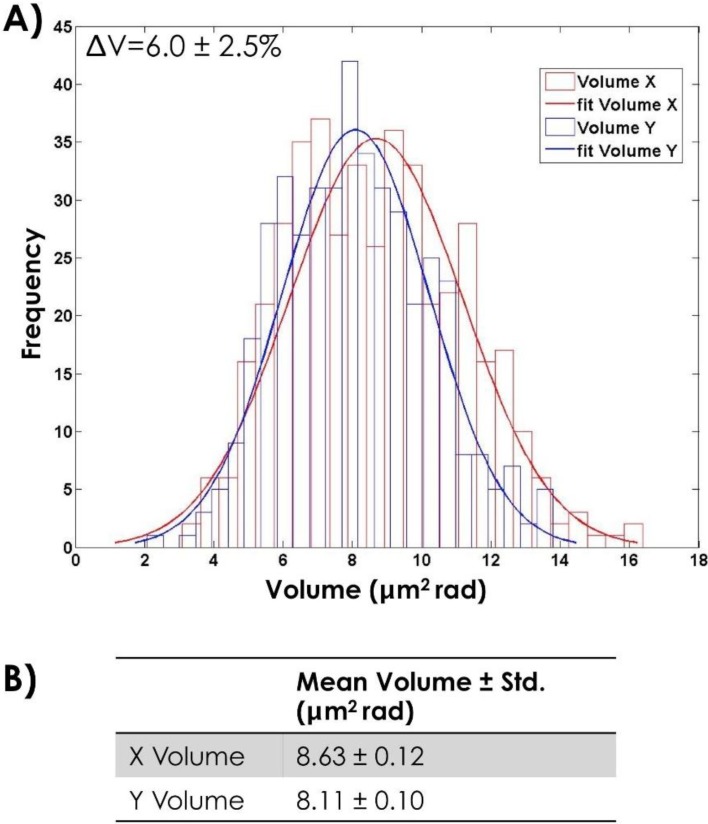
(**A**) Histogram of the measured head volume for both 500 X- and 500 Y-bearing sperm cells and the correspondent fit; (**B**) Mean value of the head volume obtained by the fit.

Measures of the volume of sperm head are performed on 500 X- and 500 Y-bearing sperm cells from two different bulls to take into accounts the variation from cell to cell and bull to bull. Results are reported in Figure 8A and summarized in Figure 8B. It is evident that, even if the mean value of the volume is bigger for X-bearing sperm cells respect to Y-bearing sperm cells, as expected, the X- and Y-Gaussian are widely overlapped. Indeed, considering that the standard deviation in volume differences (2.5%) is comparable with the differences in DNA content (3.8% in bull sperm), these parameters do not allow the unequivocal identification of the two cell populations. The label-free identification of DNA content, plasma-membrane proteins or biochemical signatures for either X- or Y-bearing sperm cells would offer great opportunities for separation.

**Figure 9:**
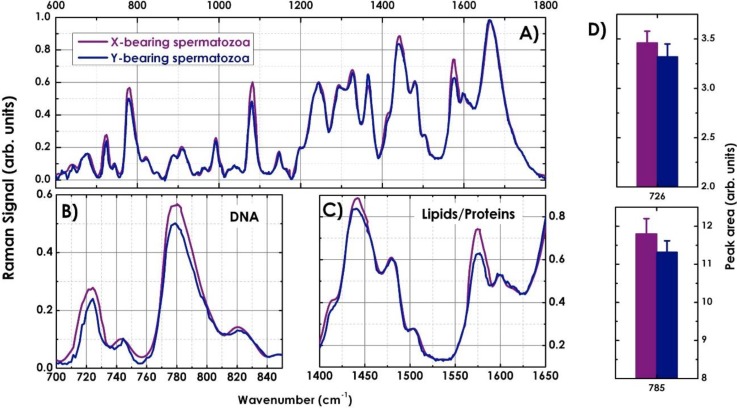
(**A**) Average Raman spectra of 300 X- (purple line) and 300 Y-sperm cells (blue line) in the “fingerprint” spectral region; (**B**) Comparison between the Raman spectra of X- and Y-spermatozoa in the spectral region between 700–850 cm^−1^ and (**C**) 1400–1650 cm^−1^. (**D**) Measured peak area of the characteristic DNA bands at 726 and 785 cm^−1^ for X- and Y-spermatozoa.

Figure 9A shows typical Raman spectra, between 600 and 1800 cm^−1^, of X- (purple line) and Y-bearing sperm cells (blue line) of a bull acquired from the nucleus region. Each spectrum is an average of 300 cells. Just before the acquisition the sample autofluorescence was bleached exposing the cell for about 40 s to the laser light and Raman spectra were recorded using an integration time of 20 s. The spectra were corrected by subtracting the background spectrum (quartz slide and PBS solution) and normalized. The acquired spectra are like molecular fingerprints representing contributions from various cellular components such as DNA, protein, lipids and carbohydrates, and a summary of the wavenumbers and their corresponding band assignment is given in Table 1. The sperm spectra show the characteristic cell features: a strong Amide I band around 1660 cm^−1^, an intense CH deformation band around 1450 cm^−1^, as well as the sharp band at 1005 cm^−1^ assigned to the amino acid phenylalanine. The two spectra look very much alike, however, X- and Y-spermatozoa vary in their composition and therefore also in their Raman spectra. The X-bearing spermatozoa show increased intensity of the peaks at 726, 785 and 1581 cm^−1^ (see Figure 9B,C), that are assigned to ring breathing modes in the DNA bases, as well as in the 1095 cm^−1^ mode of the symmetric ${\text{PO}}_{2}^{-}$ stretching vibration of the DNA backbone. These features can be attributed to slightly higher DNA concentration in X- than in Y-bearing sperm cells. An additional difference between the two population of spectra and be observed in the spectral regions between 1400 and 1650 cm^−1^ (see Figure 9C) mainly assigned to the protein content. This is probably due to the presence of HY antigen absent on the membrane of X-sperm cells. Peak area measures of Raman bands at 726 and 785 cm^−1^ performed on 300 X- and 300 Y-spermatozoa are reported in the histograms of Figure 9D. More precisely, the measured mean variation of the Raman band areas is ∆A = 4.1% ± 0.4%, which is in good agreement with the expected differences in DNA content (3.8% in bull sperm).

**Figure 10:**
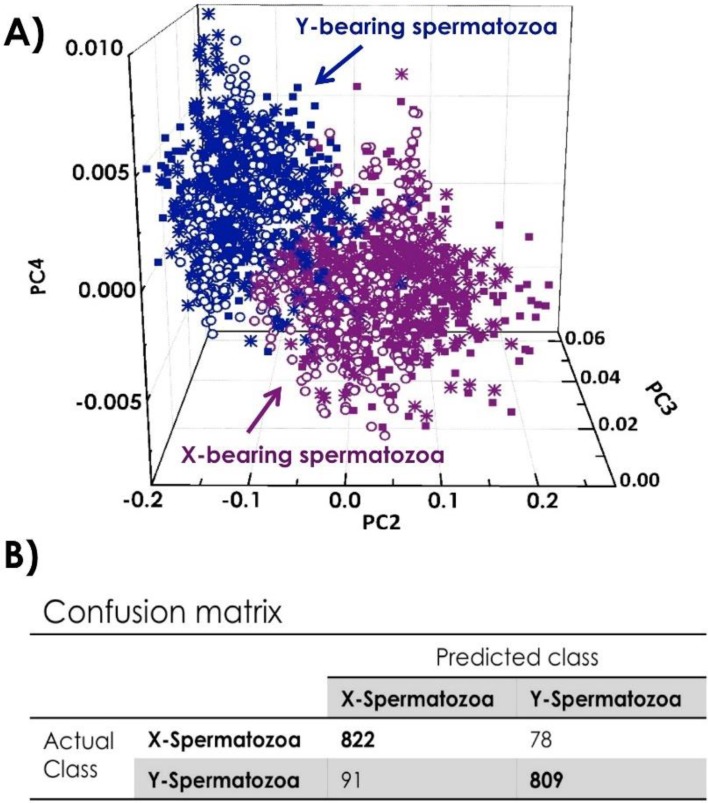
(**A**) 3D Principal Component Analysis (PCA) score plot comparing 900 X- and 900 Y-spermatozoa from 3 bulls; (**B**) Confusion matrix giving the classification for X-and Y-spermatozoa.

Principal component analysis (PCA) was used to visualize spectral differences and cluster formation according to the cell type. Figure 10A shows the 3D score plot on 1800 spectra, 300 Raman spectra of X- and Y-spermatozoa from three different bulls, where the principal components PC2, PC3 and PC4 are plotted against each other. Each cell type is clearly separated from the other one as indicated by the different marked and colored scores in Figure 10A. The first principal component PC1 is not included in the data classification as essentially due to the background variation and not directly attributed to the cell differences. PC2, PC3 and PC4 loadings reveal the most feature-rich plot [3]. Particularly evident are the peaks in the spectral regions around 780, 1100 and 1580 cm^−1^ that match the DNA vibrational modes, and around 1350–1450 cm^−1^, revealing the different contribution of the sex-associated membrane proteins in the two cell types [3]. In order to discriminate and assign X- and Y-spermatozoa, a confusion matrix was built up using the leave-one-out classification approach [4,22]. The details of the prediction for the individual cell types are depicted in Figure 10B: 1631 out of 1800 spectra could be classified correctly. This results in the high prediction accuracy of 90.2%.

## 5. Conclusions

In this work a full label-free characterization of bovine sperm cells was performed. For this purpose, an experimental set-up combining digital holography (DH) microscopy and Raman spectroscopy (RS) has been developed.

High-resolution images of bovine sperm have been obtained by DH microscopy from the reconstruction of a single acquired hologram, highlighting in some cases morphological alterations. Quantitative 3D reconstructions of sperm head, both normal and anomalous, have been studied and a “protuberance” of the head post-acrosomal region has been highlighted. In order to identify the structure and the corresponding biochemical constituents of the sample, we recorded the Raman image for the sperm cell. The Raman image confirmed the presence of the “protuberance” in the sperm post-acrosomal region, and suggested the protein vibrations as associated Raman marker of the defect. This is probably due to the presence of centrioles in the sperm region connecting the tail to the head.

Finally, the same set-up has been used to identify X and Y-chromosome-bearing sperm cells. DH imaging approach is entirely based on a possible difference in sperm head size and volume due to the variation in the amount of DNA, as the X chromosome being larger that the Y chromosome. Unfortunately, these physical parameters, as previously demonstrated by using other experimental approaches [20,45], do not allow the unequivocal identification of the two cell populations. On the other hand, the label-free identification of DNA content, plasma-membrane proteins or biochemical signatures achieved with RS allowed to non-invasively identify between X- and Y-chromosome-bearing sperm cells with a high accuracy (>90%).

The current work sets the foundation for future studies and supports continued investigation of DH-RS combined approach for sperm cell analysis and sperm disorder diagnosis in bulls, in animals and even in humans. Alternative experimental configurations, such as the implementation in microfluidic environments [14], the adoption of compressive methods [32,46] and improving data analysis [22], will enable a high degree of automation, analyzed cell number and sensitivity/specificity that is required to improve the throughput of the approach.
